# Abstracts and Selections

**Published:** 1905-10

**Authors:** 


### Abstracts and Selections.

Much as we deplore the suffering and loss of life in the prevailing yellow fever epidemic of Louisiana, it seems providentially sent with a message, and if this message be properly interpreted by the medical profession, results will follow making the epidemic no unmitigated curse. The good to be accomplished may be divided into three general heads. In the first place, we are promised that the city of New Orleans shall be thoroughly cleaned. This means the saving of many lives from other diseases than yellow fever. It means another American municipality getting a lesson in public health, the only sort of lesson they ever seem to heed.

A second result will be the popular appreciation of what the work of General Leonard Wood and his medical associates in Cuba meant, and what the present results in Panama are. The average number of deaths per day in the Louisiana epidemic has been greater than the average per month in the Canal Zone under American control.

Finally, what more forcible argument for a National Board of Health can be brought than the present "shot-gun quarantine" of Louisiana by her sister State? It deserves a place beside Lynch law and the Vigilance Committees of horse-theft days on the frontier. It is to be hoped that the legislative committee of the American Medical Association who are presenting to Congress the need of a National Board of Health, will not fail to push home the lesson of this epidemic. We have national commissions to regulate interstate commerce, immigration, and a host of things. We have a whole department of the government with a cabinet officer devoted to the care of our agriculture, making constant warfare on the parasites of our plant life. But when a human parasite, like the yellow fever germ, begins to devastate *human* life the National government is powerless and each man must stand before his own home with a shotgun, while sister States of this Union are arrayed against each other with armed troops on their borders by order of the Governors of the States. It should be pointed out in this connection, also, that this sort of epidemic control is as ineffective as it is crude because the mosquito, the real bearer of the disease, can not be kept away by a shotgun.—*Detroit Medical Journal.*

---

THE American Anti-Tuberculosis League, which held a much-heralded meeting at Atlanta, Ga., and decided to hold its next meeting at El Paso, Texas, is having troubles of its own. The President has resigned, and Dr. H. N. Crouse, the secretary of the local committee at El Paso, has resigned, and it was discovered that the League has no legal existence. Dr. W. N. Vilas, the Secretary, says he knows nothing of the nature of the organization.—*Southern California Practitioner.*

This League is the outgrowth of the bolters' movement in 1902, when the American Congress on Tuberculosis, jointly with the Medico-Legal Society, held its third annual meeting in New York. The bolters organized the seceders, with Daniel Lewis as President and George Brown, of Georgia, as Secretary, and appropriated the name of the parent society. Lewis resigned and Brown was made President, and the name was changed. The "Brown Congress" held a meeting in Atlanta last spring and Brown was presented with a watch. An Iowa man—name forgotten—was made President and Dr. Vilas, of El Paso, Secretary. The next meeting was appointed for El Paso, by invitation of citizens and physicians. There is no connection whatever between this organization and the American International Congress on Tuberculosis which had such a successful meeting at St. Louis last October, and which will meet again in 1906.

